# Supplementary material for: Targeting myelin lipid metabolism as a potential therapeutic strategy in a model of CMT1A neuropathy
Source: Nat Commun. 2018 Aug 2;9:3025. doi: 10.1038/s41467-018-05420-0 (PMC6072747; doi:10.1038/s41467-018-05420-0)
Supplement: Supplementary file 1 — Supplementary Information [file 41467_2018_5420_MOESM1_ESM.pdf]

Supplementary Information

Targeting myelin lipid metabolism as a potential therapeutic strategy in a model of CMT1A neuropathy

by Fledrich and Abdelaal *et al*.

Supplementary Figure 1:

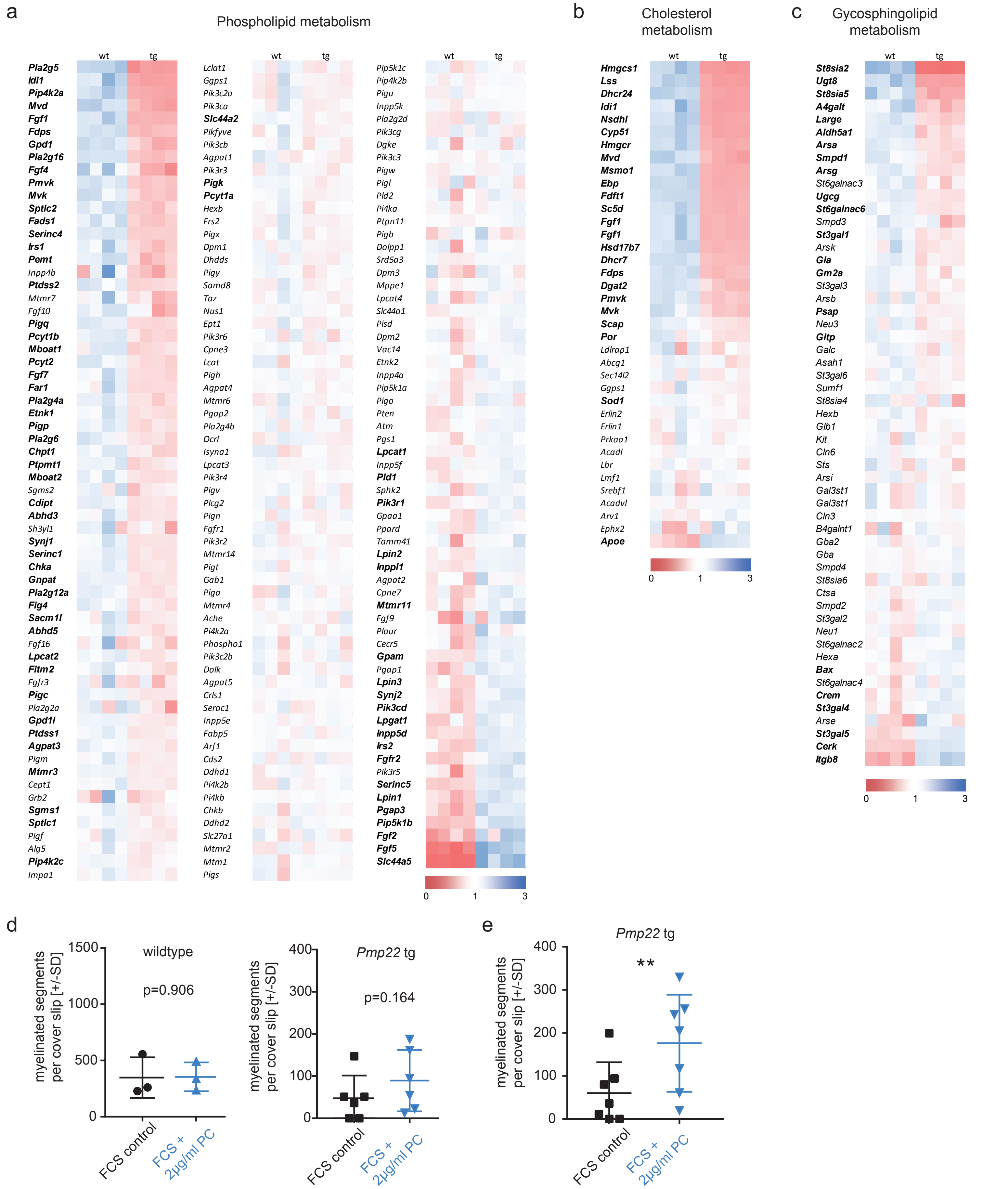

Supplementary Figure 1: Perturbed lipid metabolism in Pmp22tg Schwann cells.
a-c Gene Ontology (GO) gene sets for the major myelin lipid classes were used to visualize alterations between wildtype (Wt, n=4) and CMT rat (Tg, n=4) mRNA expression in sciatic nerve at P18. The gene sets comprise (**a**) phospholipid biosynthetic process (M11978), (**b**) regulation of cholesterol biosynthesis and metabolism (M12147 and M15312) and (**c**) glycosphingolipid biosynthetic process and metabolism (M13299 and M13858). The gene identifier is given to left of each color coded expression and bold indicates statistically significant regulation.
d Wt (left, n=3) and *Pmp22*tg (right, n=6) DRG cocultures grown in full serum media treated with 2µl/ml PC for 10 days after myelination induction display no treatment effect. Two cover slips per embryo were generated, respectively, whereas one culture was treated and the other culture served as control (paired T-test).
e Pmp22tg DRG cocultures grown in delipidated media treated with 2µl/ml PC for 10 days after myelination induction displays improved myelination after treatment, as assessed by immunocytochemical quantification of MBP positive myelin segments per culture. In contrast to Figure 1e, two cover slips per embryo were generated and one culture was treated and the other culture served as control (n=7, paired T-test, p-value: **<0.01; mean +/- standard deviation (SD)).

Supplemental Figure 2

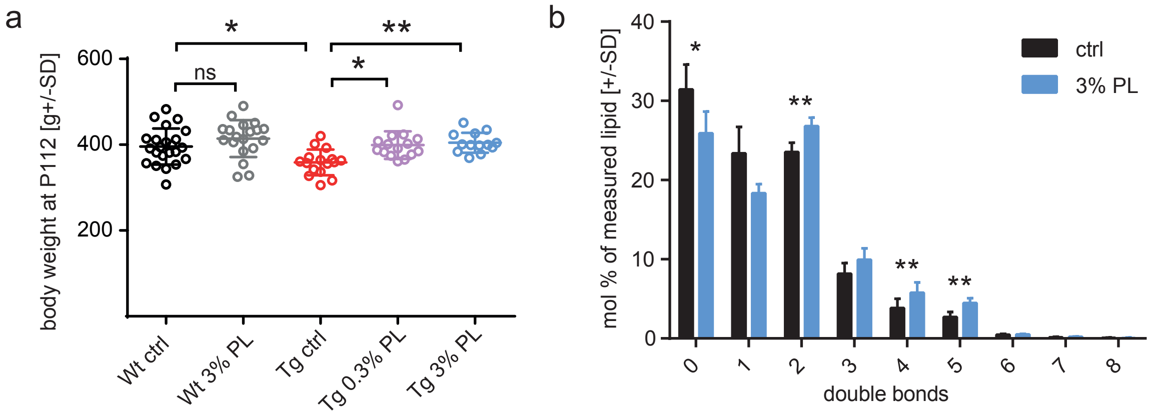

Supplementary Figure 2: Dietary phospholipid therapy with CMT rats.
a The body weight of wildtype and CMT rats at P112 after PL treatment from P2-P112 revealed no weight gain in treated (3%PL, n=19) compared to control (n=23) wildtype rats. Body weight is significantly lower in control CMT rats (n=16) compared to wildtype controls, and again increased after PL treatment CMT rats (0.3%, n=15; 3%, n=13), which may reflect preservation of muscle mass (see Figure2D), (one-way ANOVA, Tukey's post test)
b Mass spectrometric analysis of the stomach content (milk) of P9 pups, that were nursed by 3% PL treated (blue, n=6) and nontreated (black, n=5) dams from P2-P9 (milk per litter was pooled). Plot shows all neutral lipids (>98% TAG and DAG) according to their double bonds. Significant shift towards species with either two or four double bonds is visible, indicating that the supplemented phospholipid fatty acid tails (mostly C18:2) have passed the mothers milk in the form of neutral lipids. Adjusted p-values are shown (One-way ANOVA, p-value: **<0.01, *<0.05).

Supplemental Figure 3

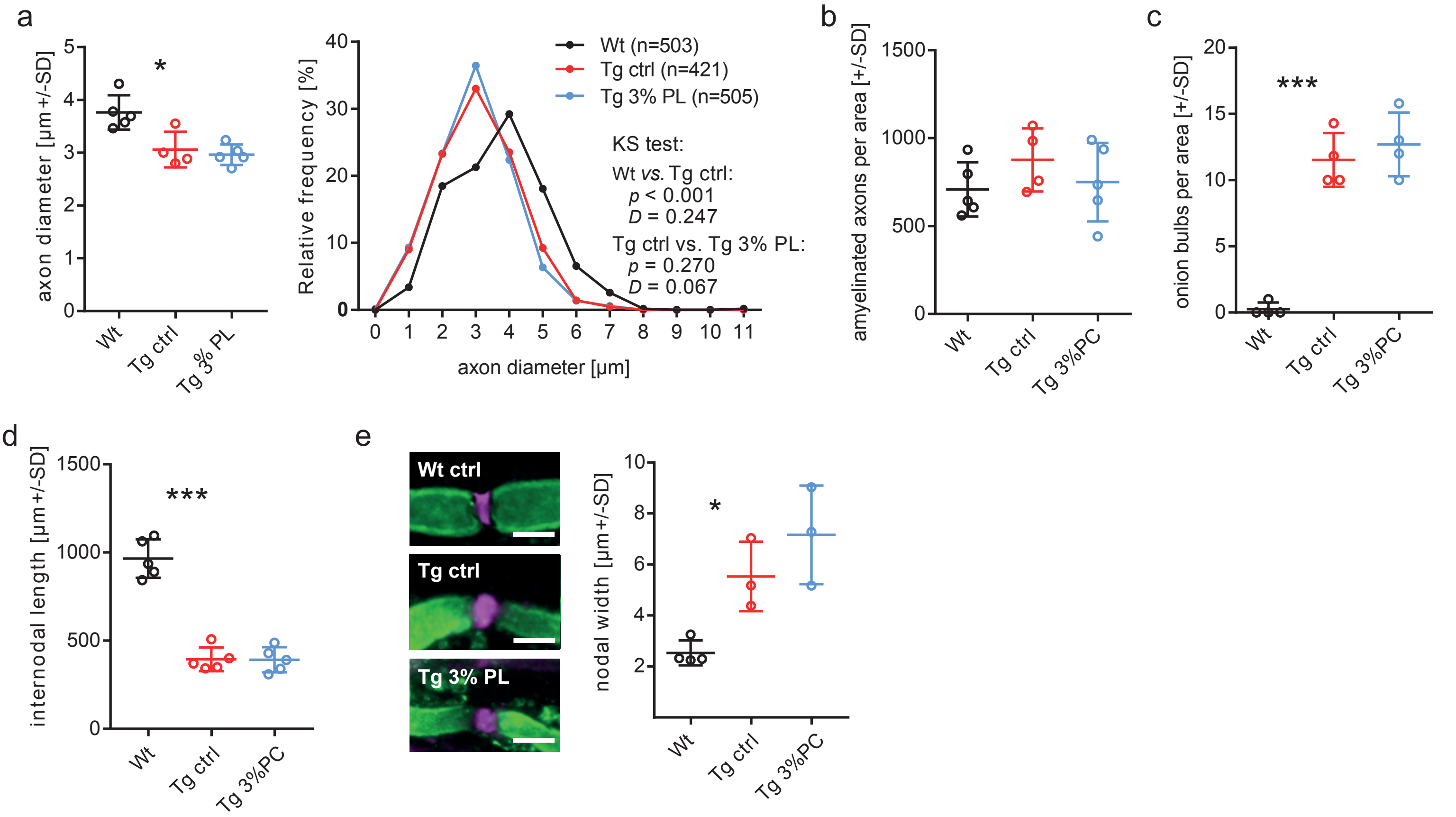

Supplementary Figure 3: Peripheral nerve morphometry after phospholipid therapy.

a Electronmicroscopic quantification of the axonal diameter in tibial nerve cross sections at P112 as extracted from the g-ratio analyses in **Figure 4A** revealed reduction in Tg ctrl rats (red, n=4) compared to Wt (black, n=5), but no alteration upon 3% PL treatment (blue, n=5). 82 to 110 fibers per animal were measured (left panel: one way ANOVA with Tukey's post test, p-value: *<0.05; right panel: percentage distribution blot with KS-Test).

b Electronmicroscopic quantification of the number of physiological unmyelinated axons (<1µm) in tibial nerve cross sections per area at P112 revealed no difference between Tg ctrl rats (red, n=4) compared to Wt (black, n=5), and no alteration upon 3% PL treatment (blue, n=5; One way ANOVA with Tukey's post test; mean +/- standard deviation [SD]).

c Electronmicroscopic quantification of the number of onion bulbs per area at P112 revealed significant increase in Tg ctrl rats (red, n=4) compared to its virtual absence in Wt (black, n=5), and no alteration upon 3% PL treatment (blue, n=5). One way ANOVA with Tukey's post test.

d,e Immunohistochemical analysis of teased fiber preparations from P112 tibial nerves revealed shortened internodal length (**d**) and increased nodal width (**e**, NaV1.6, magenta; MBP, green) in Tg ctrl when compared to Wt, but no alteration in both parameters after 3% PL therapy (n=5 per group; one way ANOVA with Tukey's post test, p-value: *<0.05, ***<0.001).

Supplemental Figure 4

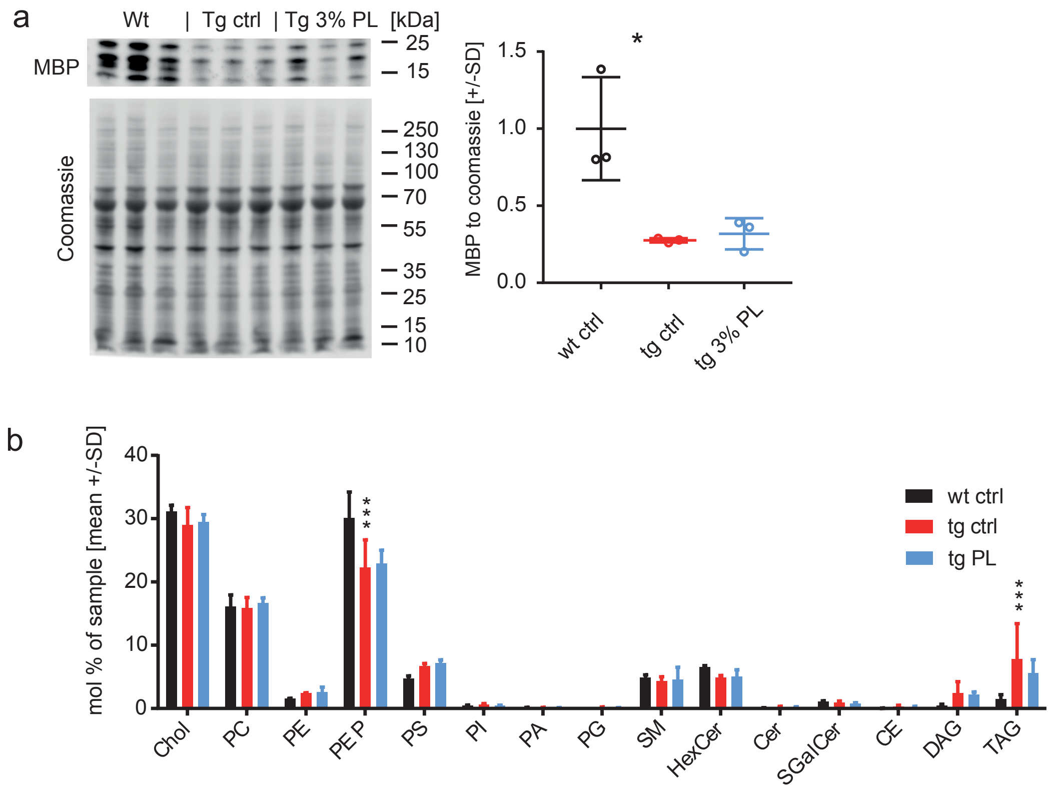

Supplementary Figure 4: Molecular analyses of pheripheral nerves after phospholipid therapy
a Western blot analysis of MBP from P112 sciatic nerve full protein preparations from Wt, Tg ctrl and Tg 3% PL treated (P2-P112) treated rats. As loading control, a coomassie staining of the gel was performed (n=3 per group, left panel). Quantification (right panel) shows decreased MBP abundance in Tg compared to Wt nerves). In non-treated (Tg ctrl) versus treated (Tg 3%PL) CMT rats, no quantitative difference of MBP abundance could be detected, when normalized to Coomassie (n=3 per group, One way ANOVA with Tukey's post test; p-value: *<0.05; mean +/- standard deviation [SD]).
b Mass spectrometric analysis of sciatic nerve myelin from Wt ctrl (black, n=4), Tg ctrl (red, n=3) and Tg PL (blue, n=3) rats, purified after study end (P112), normalized to total lipid shows widely unaltered stoichiometry (as mol percent) between Wt, Tg ctrl and Tg PL, except lower PEP and higher TAG in CMT rats compared to Wt. Chol: cholesterol, PC: phosphatidylcholine, PE: phosphatidylethanolamine, PEP: PE plasmalogens, PS: phosphatidyl-serine, PI: phosphatidylinositol, PA: phosphatidic acid, PG: phosphatidylglycerol, SM: sphingomyelin, HexCer: hexylceramide, Cer: ceramide, SGalCer: S-galactosylceramide, CE: cerebroside, DAG: diacylglycerol, TAG: triacylglycerol. Adjusted p-values are shown (two-way ANOVA).

Supplemental Figure 5

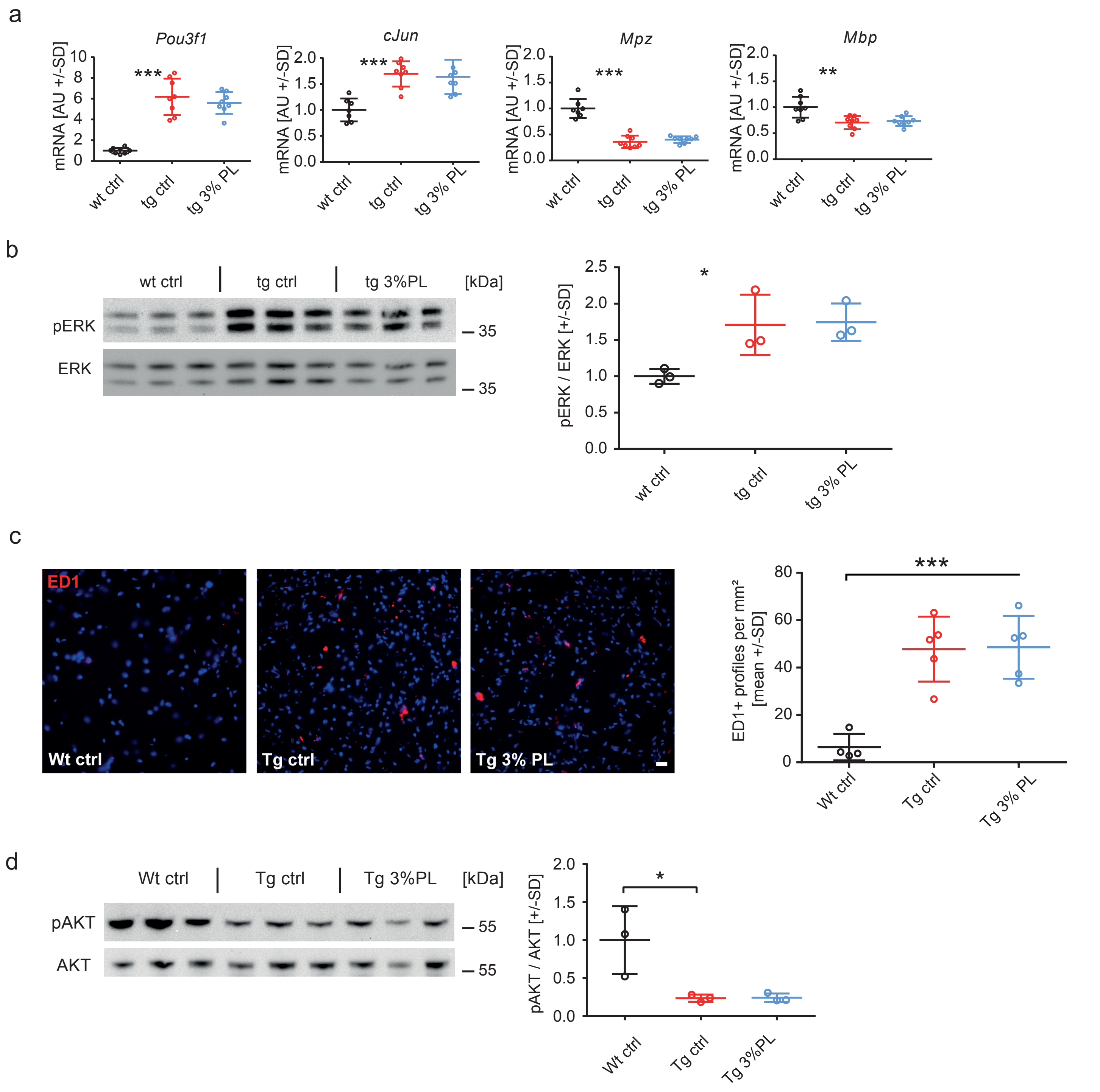

Supplementary Figure 5: Unaltered Schwann cell differentiation after phospholipid therapy.

a qPCR analysis with sciatic nerve mRNA extracts of P112 wildtype rats (wt), non-treated CMT rats (tg ctrl) and CMT rats treated from P2-P112 with 3% PL. No treatment effect can be detected for the differentiation markers Pou3f1, Mpz, Mbp and the dedifferentiation marker cJun (n=8 per group, One way ANOVA with Tukey's post test; p-value: *<0.05, **<0.01, ***<0.001; mean +/- standard deviation [SD]).

b Western blot analyses with full sciatic nerve lysates from P112 Wt ctrl, Tg ctrl und Tg 3%PL (P2.P112) rats shows no amelioration of the increased ERK activity in CMT rats after PL treatment, as assessed by ERK phosphorylation over constitutive ERK. Quantification is shown in the right panel (n=3 per group, One way ANOVA with Tukey's post test).

c Phospholipid therapy in CMT1A rats from P2-P112 does not influence the number of activated macrophages. Immunohistochemistry against activated macrophages (ED1) in tibial nerve cross sections from a Tg ctrl and Tg rat treated with 3% PL at P112 (left panels). Representative micrographs are shown. Scale bar, 20µm. Quantification (right panel) shows similar numbers of activated endoneurial macrophages (ED1-positive profiles/mm2) in Tg ctrl rats (red, n=5) when compared to Tg rats treated with 3% PL (blue, n=5, One way ANOVA with Tukey's post test).

d Western blot analysis with full nerve lysates from Wt, Tg ctrl and Tg 3%PL (P2-P21) treated rats was performed at P21 and shows less AKT activity in Tg rats as measured by phosphorylated AKT over constitutive AKT. No alteration was revealed after PL treatment. Quantification is shown in the right panel (n=3 per group, One way ANOVA with Tukey's post test).

Supplemental Figure 6

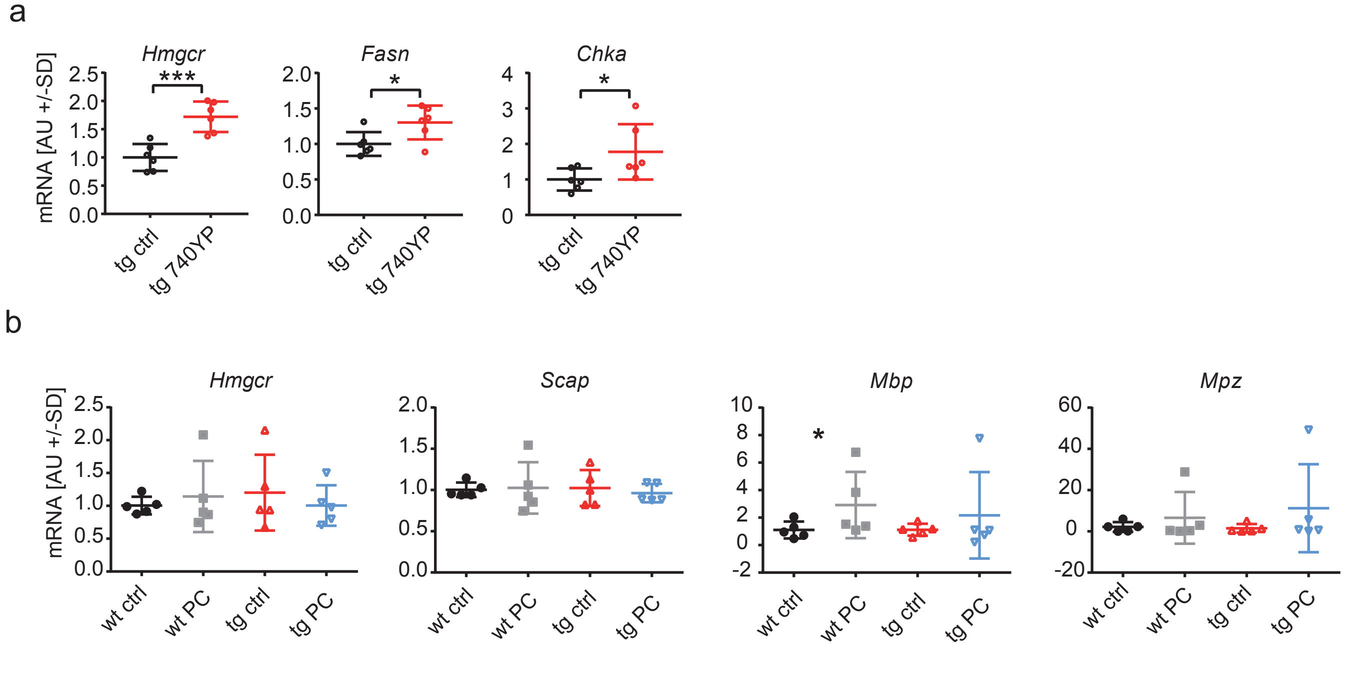

Supplementary Figure 6: Myelin gene expression in Schwann cells *in vitro*.
a In purified primary Schwann cells from CMT rats, the treatment with a specific PI3K activator (740YP) resulted in an increased mRNA expression of the lipid genes Hmgcr, Fasn and Chka, as assessed by qPCR 6h after treatment began (n=6 per group; Student's T test; p-value: *<0.05, **<0.01, ***<0.001; mean +/- standard deviation [SD]).
b Dorsal root ganglia neuron and Schwann cell cocultures from wildtype (Wt) and Pmp22 transgenic (Tg) mice grown in delipidated media either treated as control or with 2µg/ml PC as in **Figure 1e**. Ten days after myelination induction, cultures display virtually no difference in the mRNA expression of the cholesterol genes Hmgcr and Scap, as well as of Mpz. A slight increase in wildtype cocultures could be measured for Mbp (n=5 individual cultures per group, one way ANOVA with Tukey's post test).
