# Supplementary material for: Targeting myelin lipid metabolism as a potential therapeutic strategy in a model of CMT1A neuropathy
Source: Nat Commun. 2018 Aug 2;9:3025. doi: 10.1038/s41467-018-05420-0 (PMC6072747; doi:10.1038/s41467-018-05420-0)
Supplement: Supplementary file 3 — Description of Additional Supplementary Files [file 41467_2018_5420_MOESM3_ESM.pdf]

Description of Additional Supplementary Files

File Name: Supplementary Data 1
Description: Table showing overlap of internal standards and endogenous lipid species. Lipid extractions were performed in the absence of internal lipid standards. Mass spectrometry and data evaluation was performed as described in Methods. Intensities monitored at m/z values corresponding to standards are normalized to the sum of peak intensities monitored for the respective lipid class. Data are presented as % intensities of n=4-6 measurements.

File Name: Supplementary Data 2
Description: PCR primer sequences.

File Name: Supplementary Data 3
Description: Overview of all RNA-seq samples with detailed information about condition, tissue and mapping statistics. Note that "Uniquely Mapped Reads" refers to uniquely mapped to transcript.
